# Cross-Sectional Survey of Clinical Trials of Stem Cell Therapy for Heart Disease Registered at ClinicalTrials.gov

**DOI:** 10.3389/fcvm.2021.630231

**Published:** 2021-07-08

**Authors:** Rong Yang, Yonggang Zhang, Xiaoyang Liao, Ru Guo, Yi Yao, Chuanying Huang, Li Qi

**Affiliations:** ^1^Department of International Medical Center/Ward of General Practice, West China Hospital, Sichuan University, Chengdu, China; ^2^Department of Periodical Press and National Clinical Research Center for Geriatrics, West China Hospital, Sichuan University, Chengdu, China

**Keywords:** stem cell, heart disease, therapy, cross-sectional survey, ClinicalTrials.gov

## Abstract

**Objective:** It is important to register clinical trials before their implementation. There is a lack of study to evaluate registered clinical trials of stem cell therapy for heart diseases. Our study used the registration information at ClinicalTrials.gov to provide an overview of the registered trials investigating stem cell therapy for heart diseases.

**Methods:** We searched ClinicalTrials.gov from inception to October 1, 2020 to identify clinical trials evaluating stem cell therapy for heart diseases. These trials were included in a cross-sectional survey and descriptive analysis. The outcomes included start date, completion date, location, status, study results, funding, phase, study design, conditions, interventions, sex, age, and sample size of those trials, as well as conditions, efficacy, safety and samples of the publications. SPSS 24.0 software was used for the statistical analysis.

**Results:** A total of 241 trials were included. The registration applications for most trials originated from the United States, and the research start date ranged from 2001 to 2025. More than half of the trials have been completed, but few trials have published results (15.62%). The funding source for 81.12% of trials was recorded as “other” because the specific funding source was not indicated. There were 226 (93.78%) interventional studies and 15 (6.22%) observational studies; among all 241 studies, only 2.90% were phase 4 trials. Most interventional studies used randomized allocation, parallel assignment, and blinding. Of the observational studies, 6 were cohort studies (40.00%) and 73.33% were prospective. The most common disease was coronary artery disease (57.68%) and 98.34% included both male and female participants. The sample size included fewer than 50 patients in 58.51% of trials, and only 18 trials (7.47%) lasted more than 121 months. The registered details were illogical for nine trials (3.8%) that included 0 subjects and two trials (0.8%) that had a duration of 0 months (0.8%). In term of publications of the trials, most of the publications of the trials showed efficacy and safety in stem cell therapy for heart disease.

**Conclusion:** The clinical trials investigating stem cell therapy for heart diseases registered at ClinicalTrials.gov are mostly interventional studies, and only a few are phase 4 trials. Most trials have a small sample size, and few have a duration of more than 121 months. Most of the completed trials did not publish their results, and some of the registration information was incomplete and illogical.

## Introduction

Under United States federal law, the National Institutes of Health has been required since 1997 to create public resources providing information about clinical trials regulated by the United States Food and Drug Administration^1^. Clinical trial registration and result publication have multiple benefits. For individuals, this information can help patients understand their condition and the latest treatment plan and progress; for the general public, knowledge about clinical trials can eliminate unnecessary anxiety and better promote population health; for researchers and institutions, this information can reduce unnecessary repeated trials, reduce the risk of bias, and increase the reliability of trial results (1). Therefore, it is important for the progress and results of clinical trials to be announced in a timely and truthful manner.

Cardiomyocytes are terminally differentiated cells that cannot differentiate to repair and remodel the damaged heart (2, 3). In contrast, stem cells have immortal self-renewal ability and can produce at least one type of highly differentiated progeny cell (3). Therefore, stem cell therapy shows potential in damage repair and cure of refractory diseases, such as spinal cord injury (4), nerve pain (5), and immune regulation (6). Stem cell therapy is also a promising treatment for heart diseases. Stem cell therapy effectively improves cardiac ejection fraction and reverse remodeling in heart failure (7), improves the survival rate, exercise capacity, and performance of patients with non-ischemic cardiomyopathy (8), and reduces the onset of refractory angina (9). However, the Efficacy and safety of stem cell therapy for heart diseases are unclarified, with potential risks of tumorigenicity, immunogenicity, and arrhythmia (10). Rigorously designed clinical trials are needed to prove the Efficacy of stem cell therapy in treating heart diseases and improve technology to reduce the related risks. Clinical trial registration is an important step in making these clinical trials open and transparent, reducing redundant research, and identifying future research directions (11). Therefore, we analyzed the characteristics of trials registered at ClinicalTrials.gov that investigated stem cell therapy for heart diseases, aiming to provide a comprehensive overview of this topic for individuals, the public, researchers, and institutions.

## Methods

### Platform Selection

ClinicalTrials.gov is a clinical trial database founded in 1997 that is jointly operated by the National Library of Medicine and the United States Food and Drug Administration. Up to now, 216 countries from 50 regions around the world have registered trials at ClinicalTrials.gov. As of October 1, 2020, there were 353,838 trials registered at ClinicalTrials.gov. Therefore, the ClinicalTrials.gov database was selected as the source from which data was collected for the present analysis.

### Data Extraction

We performed an advanced search of ClinicalTrials.gov. First, we searched the database for trials that included the PubMed MeSH terms “heart disease” and “stem cell,” and performed a radiation search by matching each term with “condition or disease” and “intervention/treatment,” respectively. We retrieved relevant trials that were registered from database inception to October 1, 2020. For all retrieved trials, we recorded the unique identification code (NCT number), title, start date, completion date, location, status, study results, funding, phase, study design, conditions, interventions, outcome measures, sex, age, sample size, and uniform resource location (URL). Duplicates and irrelevant trials were eliminated. We also excluded trials in which the heart diseases were relieved by the treatment of diseases in other body systems, such as treating pulmonary fibrosis to reduce pulmonary hypertension and treating kidney disease to improve heart function. If there was unclear or missing information, the researchers entered the URL and NCT number into the database to confirm and supplement this information. After that, we searched articles by NCT numbers for analyzing efficacy and safety of stem cell therapy. All abovementioned steps were independently completed by two researchers. Any controversy was resolved by a third researcher.

### Statistical Analysis

Data on the location, start date, status, study results, and funding were recorded to provide a basic overview of the registered trials. Research type, allocation, blinding, and other information were used to describe the study design. Conditions, sex, age, sample size, and study duration were used to show specific research content. Finally, conditions, efficacy, safety and samples were recorded to analyze the articles. Categorical variables were described as frequency and percentage, whereas continuous variables were described as the maximum, minimum, median, and average.

## Results

**Figure 1:**
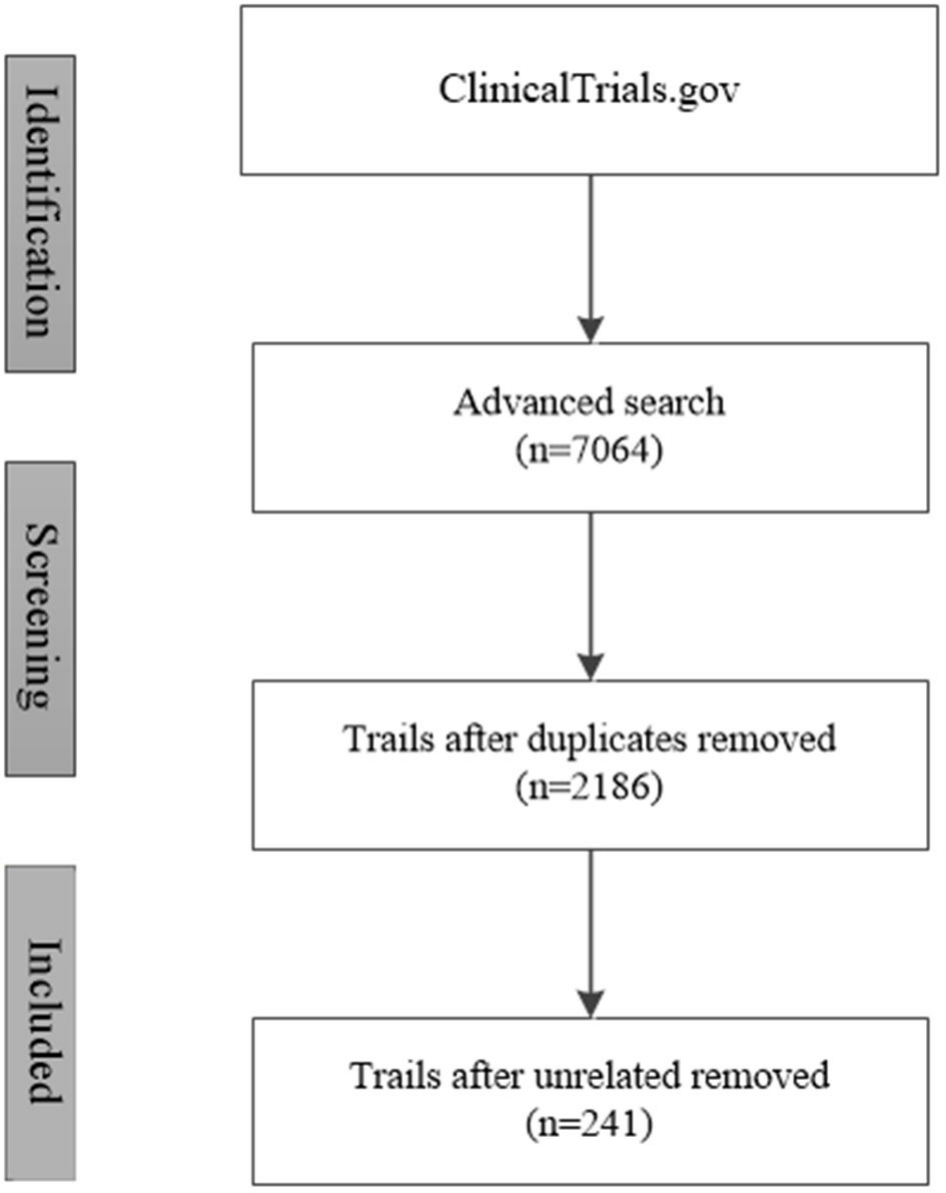
Trial retrieval process.

The initial search retrieved 7,064 clinical trials. Among them, 4,878 trials were repeated, and 1,945 were not related to the aims of the present study. A final total of 241 trials were included (Figure 1).

### Basic Overview

#### Location

The 241 included trials were performed in 35 countries. The five most common countries were the United States (67 trials; 27.80%), China (20 trials; 8.30%), Germany (13 trials; 5.39%), Denmark (12 trials; 4.98%), and France (12 trials; 4.98%). Classification in accordance with the continent of origin showed that most trials were conducted in Europe (34.85%) and North America (31.54%), followed by Asia (21.99%), South America (4.15%), and Oceania (0.41%). The location was not stated for 17 trials (7.05%). The distribution of the trial locations is shown in Table 1.

**Table 1:** Location of trials.

	**Absolute (*n* = 241)**	**Relative (%)**
**North America**	76	31.54%
United States	67	27.80%
Mexico	4	1.66%
Canada	5	2.07%
**South America**	10	4.15%
Brazil	8	3.32%
Colombia	1	0.41%
Chile	1	0.41%
**Asia**	53	21.99%
China	20	8.30%
Japan	8	3.32%
Iran	6	2.49%
Korea	5	2.07%
India	4	1.66%
Israel	4	1.66%
Kazakhstan	2	0.83%
Indonesia	2	0.83%
Malaysia	1	0.41%
Singapore	1	0.41%
**Europe**	84	34.85%
Germany	13	5.39%
Denmark	12	4.98%
France	12	4.98%
Spain	10	4.15%
United Kingdom	6	2.49%
Italy	6	2.49%
Slovenia	5	2.07%
Russian	4	1.66%
Belgium	3	1.24%
Poland	3	1.24%
Greece	2	0.83%
Austria	2	0.83%
Finland	2	0.83%
Ukraine	1	0.41%
Switzerland	1	0.41%
Norway	1	0.41%
Turkey	1	0.41%
**Oceania**	1	0.41%
Australia	1	0.41%
**Unknown**	17	7.05%

#### Start Date

The earliest start date was 2001 and the latest was 2025(not begin), with only one trial started in each of these years. Most trials were started in 2007 (20 trials; 8.30%), followed by 2008 (19 trials; 7.88%), and 2005 and 2013 (each with 18 trials; 7.47%). The distribution of the trial start date is shown in Table 2.

**Table 2:** Trial start dates.

**Start year**	**Number of trials (%)**
2001	1 (0.41%)
2002	2 (0.83%)
2003	4 (1.66%)
2004	11 (4.56%)
2005	18 (7.47%)
2006	15 (6.22%)
2007	20 (8.30%)
2008	19 (7.88%)
2009	12 (4.98%)
2010	16 (6.64%)
2011	17 (7.05%)
2012	14 (5.81%)
2013	18 (7.47%)
2014	12 (4.98%)
2015	12 (4.98%)
2016	10 (4.15%)
2017	8 (3.32%)
2018	8 (3.32%)
2019	11 (4.56%)
2020	12 (4.98%)
2025	1 (0.41%)

#### Status and Results

The status was “active, not recruiting” for 27 trials (11.20%), “recruiting patients” for 26 (10.79%), “completed” for 101 (41.91%), and “terminated” for 27 (11.20%). Three trials (1.24%) were suspended and 10 (4.15%) were withdrawn; the reasons for trial suspension and withdrawal are listed in Table 3. The status of 47 trials (19.50%) was unknown. Among the trials that had been completed or terminated, the results were only available for 20 trials (15.62%).

**Table 3:** Reasons for trial withdrawn.

**NCT number**	**Reasons**
NCT02107118	contract issues
NCT01502514	no participants enrolled
NCT01502501	no participants enrolled
NCT00279539	study never started
NCT00346177	funding not obtained and no participants enrolled
NCT00463853	inability to recruit due to administrative difficulties at the site
NCT01458405	unknown
NCT01770613	corporate business decision, may consider different subject population
NCT03272191	company dissolved
NCT01974128	no participants enrolled

### Funding

Three trials (1.20%) received United States federal funding support, 21 (8.43%) received funding support from the National Institutes of Health, 65 (26.10%) received industrial funding support, and 202 (81.12%) received funding from other sources. The funding source was not reported for eight trials (3.21%).

### Study Design

Among the 241 included trials, 226 (93.78%) were interventional studies, and 15 (6.22%) were observational studies. Fifty-two trials were phase I clinical trials (21.58%), 64 were phase 2 (26.56%), 18 were phase 3 (7.47%), seven were phase 4 (2.90%), and 49 (20.33%) were phase 1 and phase 2. There were 15 (6.22%) phase 2 and phase 3 trials, and the phase of another 36 trials (14.94%) was not applicable or unknown (Table 4).

**Table 4:** Trial phases.

**Phase**	**Number of trials (%)**
Phase 1	52 (21.58%)
Phase 2	64 (26.56%)
Phase 3	18 (7.47%)
Phase 4	7 (2.90%)
Phase 1|Phase 2	49 (20.33%)
Phase 2|Phase 3	15 (6.22%)
Not applicable or unknown	36 (14.94%)

#### Interventional Studies

In the interventional studies, the group allocation was randomized in 164 trials (72.57%), “other” in 61 (26.99%), and not applicable in one (0.4%); 147 trials (65.04%) used parallel assignment, 60 (26.55%) used single-group assignment, nine used crossover assignment, four used factorial assignment, two used sequential assignment, and the assignment in four was not applicable or unknown. A total of 127 interventional trials were blinded (35 were single-blinded, 32 were double-blinded, 20 were triple-blinded, and 40 were quadruple-blinded), 98 were non-blinded, and the blinding in one interventional trial was not applicable or unknown (Table 5).

**Table 5:** Study design of interventional studies.

**Study design**	**Absolute (*n* = 226)**	**Relative (%)**
**Allocation**		
Randomized	164	72.57%
Other	61	26.99%
Not applicable	1	0.44%
**Blinded**		
Single	35	15.49%
Double	32	14.16%
Triple	20	8.85%
Quadruple	40	17.70%
None	98	43.36%
Not applicable or unknown	1	0.44%
**Model**		
Parallel assignment	147	65.04%
Single group assignment	60	26.55%
Crossover assignment	9	3.98%
Factorial assignment	4	1.77%
Sequential assignment	2	0.88%

#### Observational Studies

Among the observational studies, there were six (40.00%) cohort studies, three (20.00%) case-only studies, one (6.67%) case-control study, one (6.67%) family-based study, and two studies (13.33%) classified as “other.” Two trials (13.33%) did not provide information on the study design. Eleven observational studies (73.33%) were prospective, one (6.67%) was retrospective, one (6.67%) was cross-sectional, and one (6.67%) was classified as “other”; two trials (13.33%) did not provide this information (Table 6).

**Table 6:** Study design of observational studies.

**Study design**	**Absolute (*n* = 15)**	**Relative (%)**
**Model**		
Cohort	6	40.00%
Case-only	3	20.00%
Case-control	1	6.67%
Family-based	1	6.67%
other	2	13.33%
unknown	2	13.33%
**Time perspective**		
Prospective	11	73.33%
Cross-sectional	1	6.67%
Retrospective	1	6.67%
other	1	6.67%
unknown	2	13.33%

### Specific Research Content

Among the 241 included trials, the most common heart condition was coronary artery disease (139 trials; 57.68%), followed by heart failure (50 trials; 20.75%) and cardiomyopathy (30 trials; 12.45%). Regarding the sex of the participants, most trials (237 trials; 98.34%) included both male and female participants; three trials (1.24%) only included male participants, and one trial did not state the sex of the participants. Most trials included adults and older adults (214 trials; 88.80%); 14 trials only included children, three only included adults, one included children and adults, and nine included children, adults, and older adults.

In terms of the sample size, the median number of registered patients was 40 (range 0–800). The number of enrolled patients was 50 or fewer in 141 trials (58.51%), 50–100 in 51 trials (21.16%), 101–150 in 19 trials (7.88%), 151–200 in 13 trials (5.39%), more than 200 in 14 trials (5.81%), and unknown in three trials.

If the research plan did not report an end-date for the trial, the end-date was set as October 1, 2020. As a result, the research duration ranged from 0 to 218 months; 10 trials ran for 12 months or less, 37 (15.35%) ran for 13–24 months, 37 (15.35%) ran for 25–36 months, 45 (18.67%) ran for 27–48 months, 42 (17.43%) ran for 49–60 months, 51 (21.16%) ran for 61–120 months, 18 (7.47%) ran for more than 121 months, and the duration was recorded as “not applicable” for one trial(Table 7).

**Table 7:** Research content.

**Characteristics**	**Absolute (*n* = 241)**	**Relative (%)**
**Conditions**		
Heart failure	50	20.75%
Coronary artery disease	139	57.68%
Cardiomyopathy	30	12.45%
Others	22	9.13%
**Gender**		
All	237	98.34%
Male	3	1.24%
Unknown	1	0.41%
**Age group**		
Child	14	5.81%
Adult	3	1.24%
Child, adult	1	0.41%
Adult, old adult	214	88.80%
All	9	3.73%
**Enrollment (*****n*****)**		
0–50	141	58.51%
51–100	51	21.16%
101–150	19	7.88%
151–200	13	5.39%
≥200	14	5.81%
Unknown	3	1.24%
**Duration (month)**		
0–12	10	4.15%
13–24	37	15.35%
25–36	37	15.35%
37–48	45	18.67%
49–60	42	17.43%
61–72	16	6.64%
73–84	20	8.30%
85–96	12	4.98%
97–108	3	1.24%
109–120	0	0.00%
≥121	18	7.47%
Unknown	1	0.41%

### Publications of Trials

The initial search retrieved 124 publications and finally retained 109 publications from 73 trials, for 13 publications were excluded for protocols, reviews and non-stem cell therapy, and 2 were excluded because of Harvard fraud stem cell investigation (NCT00474461). Among them, trial of NCT00684021 published most articles (*n* = 6), accounting for 5.50%. 68 (63.30%) articles were stem cell therapy for coronary artery disease, followed by heart failure (20 articles, 18.35%), cardiomyopathy (15 articles, 13.76%) and others (5 articles, 4.59%). 92 (84.40%) articles assessed the efficacy of stem cell therapy for heart diseases, in which 74 (67.89%) articles showed effectiveness while 18 (16.51%) showed non- effectiveness. 55 (50.46%) articles assessed the safety of stem cell therapy for heart disease with all of them showed safety. In terms of sample, 37 (33.94%) articles were <50 cases, 31 (28.44%) articles were 50 to 100 cases, 28 (25.69%) were 100–500 cases and 2 (1.83%) were more than 500 cases (Table 8).

**Table 8:** Publication status of trials.

	**Absolute (*n* = 109)**	**Relative (%)**
**Conditions**		
Heart failure	20	18.35%
Coronary artery disease	69	63.30%
Cardiomyopathy	15	13.76%
Others	5	4.59%
**Efficacy**		
Yes	74	67.89%
No	18	16.51%
Not assessment	9	8.26%
Not applicable	8	7.34%
**Safety**		
Yes	55	50.46%
No	0	0.00%
Not assessment	46	42.20%
Not applicable	8	7.34%
**Sample**		
<50	37	33.94%
50–100	31	28.44%
100–500	28	25.69%
>500	2	1.83%
Not applicable	11	10.09%

Furthermore, the articles assessed efficacy and safety in different conditions. In terms of efficacy, 10 (50.00%) articles showed effectiveness in heart failure, 46 (66.67%) showed effectiveness in coronary artery disease and 13 (86.67%) in cardiomyopathy and 5 (100.00%) in others. Fortunately, all results showed safety of stem cell therapy for heart disease if assessed (Table 9).

**Table 9:** Efficacy and safety of stem cell therapy for heart disease.

	**Heart**	**Coronary artery**	**Cardiomyopathy**	**Others**
	**failure**	**disease**		
	**(*n* = 20)**	**(*n* = 69)**	**(*n* = 15)**	**(*n* = 5)**
**Efficacy**, ***n*** **(%)**				
Yes	10 (50.00%)	46 (66.67%)	13 (86.67%)	5 (100.00%)
No	5 (25.00%)	11 (15.94%)	2 (13.33%)	0 (0.00%)
Not assessment	3 (15.00%)	6 (8.70%)	0 (0.00%)	0 (0.00%)
Not applicable	2 (10.00%)	6 (8.70%)	0 (0.00%)	0 (0.00%)
**Safety**, ***n*** **(%)**				
Yes	9 (45.00%)	40 (57.97%)	3 (20.00%)	3 (60.00%)
No	0 (0.00%)	0 (0.00%)	0 (0.00%)	0 (0.00%)
Not assessment	9 (45.00%)	22 (31.88%)	12 (80.00%)	2 (40.00%)
Not applicable	2 (10.00%)	7 (10.14%)	0 (0.00%)	0 (0.00%)

## Discussion

Heart diseases such as coronary artery disease, heart failure, and cardiomyopathy currently cannot be cured in most patients, and the complications of these heart diseases cause a great burden on patients, families, and society (12–14). Stem cell therapy provides hope for curing these heart diseases (15, 16). Because the clinical trial registration platform is a valuable resource for providing research status and tracking, we performed a cross-sectional analysis of the trials registered at ClinicalTrials.gov to comprehensively evaluate the research status of clinical trials investigating stem cell therapy for heart disease. A final total of 241 trials were included. Most trials were carried out in the United States, followed by China, Germany, Denmark, and France; these five countries are all high-ranking countries in terms of gross domestic product (GDP) (17). National GDP is positively correlated with healthcare expenditure, population health status, and life expectancy. Good population health and longer life expectancy also promote the growth of national GDP (18). Most trials were also performed in continents with a high GDP; one-third of clinical trials were conducted in each of Europe and North America. The high proportion of trials from Europe and North America may also reflect the fact that ClinicalTrials.gov is an American database, whereas other platforms are also used (19–21). The most common start date was 2007, followed by 2006–2010, and 2011–2015. Overall, the analysis shows that stem cell therapy for heart disease has attracted an increasing amount of attention in the past 20 years (4).

The present analysis of the status of registered trials evaluating stem cell therapy for heart disease found that 24.9% of trials were suspended, withdrawn, or had an unknown status; this is much higher than the 14.22% (50,301 of 353,838 trials; updated on October 9, 2020) of all trials registered at ClinicalTrials.gov. The main reason for trial withdrawal was failure to enroll patients, as well as company or enterprise decisions. Among the 128 trials that have been completed or terminated, only 20 (15.62%) have published their results on the platform. Although ClinicalTrials.gov requires clinical trials to have clear objectives, maximum transparency, and to publish results as soon as possible within a specified timeframe (22), only a few completed trials evaluating stem cell therapy for heart disease have released the results. This may be because of the relatively short period in which stem cell therapy has been has investigated for heart diseases, and the long duration of some of the related trials. Furthermore, some of the included trials may have also been registered on other platforms and published the results elsewhere. A previous study showed similar results (23).

Previous research has shown that the funding source plays a decisive role in research (24). In the present study, the funding source was listed as “other” for 202 trials (81.12%), and no funding information was listed for eight trials. Insufficient funding sources may also be the reason for the unclear status of a large number of trials and the delay in the release of the results. Therefore, it is necessary to provide more detailed funding sources in clinical trial registration to reduce the production of redundant research (25).

Of the included trials, 226 were interventional studies (93.78%) and 15 (6.22%) were observational studies (6.22%). Most were early clinical trials (phase 1, 2, 3), while only four (2.90%) were phase 4 clinical trials. This is consistent with the current related research publications and the research on stem cell therapy for heart diseases in the past 20 years (26). The current studies are still in the phase of proving the efficacy and safety of stem cell therapy for heart diseases, providing a basis for future large-scale, long-term studies. The included trials generally had good study designs, with 72.57% of intervention studies using randomized allocation, 65.04% using parallel assignment, and 56.19% using blinding. These designs are conducive to improving study quality and increasing the value of the results (27).

The main heart disease being treated by stem cell therapy was coronary artery disease (57.68%), followed by heart failure (20.75%) and cardiomyopathy (12.45%). This is because animal experiments have found that stem cells can differentiate into cardiomyocytes and coronary blood vessels (28), thereby promoting the recovery of heart structure and function (29). Based on previous animal studies, most of the patients selected for treatment had heart diseases likely to respond to stem cell therapy.

The study population comprised adults and older adults in most clinical trials (88.80%). This may be because of safety considerations, and because coronary heart disease and heart failure mostly occur in middle-aged and older adults (30, 31).

The maximum number of recruited patients was 800, and most trials (58.15%) included 50 or fewer patients. Only 5.81% of trials included more than 200 patients. This shows that there is a lack of large-scale interventional or observational research. Furthermore, some of the registered trials showed enrollment numbers of 0, which is impossible, and three trials did not include patient data. This indicates that the required clinical trial registration information was not provided. There are also problems with incomplete test information and lack of feasibility regarding the registered study duration. For example, the shortest study duration was 0 months, while only 18 trials (7.47%) ran for more than 121 months.

The publications of the trials showed that more article of stem cell therapy for coronary artery disease, which was consistent with the results of trial registered. Moreover, most of them were exploratory, most of the publications reported a conclusion that the stem cell therapy for heart disease was effective and safe. However, the registered details weakened the values of the publications.

This study is a cross-sectional analysis of registered clinical trials investigating stem cell therapy for heart diseases, and it comprehensively demonstrates the current research status of stem cell treatment for heart diseases. The main limitation of this study is that only trials registered at ClinicalTrials.gov were analyzed. Although, we searched the other clinical trials database of China which is a first-level registration agency of the world health organization's international clinical trial registration platform, while there was only 1 registered trial in this field. Another limitation is that subgroup analyses were not performed to determine whether the suspension and withdrawal of trials were dictated by the funding source, and whether the trial duration was related to the research objective and funding source. In addition, the published research results were not compared with the registered trials.

## Conclusion

This is a cross-sectional study evaluating the registered clinical trials investigating stem cell therapy for heart disease. The clinical trials investigating stem cell therapy for heart diseases registered at ClinicalTrials.gov are mostly interventional studies, with only a few phase 4 trials. Most trials have a small sample size, and few have a duration of more than 121 months. Some registered trials provided incomplete and illogical information, and there were few completed and terminated trials that provided results.

## Data Availability Statement

The original contributions presented in the study are included in the article/Supplementary Material, further inquiries can be directed to the corresponding author/s.

## Author Contributions

YZ and XL design the review. RY contributed to manuscript writing, revised, and supervised the project. RG and YY contributed to trials searches and data extraction. CH and LQ contributed to literature searches and to preparing the manuscript draft. All authors approved the manuscript.

## Conflict of Interest

The authors declare that the research was conducted in the absence of any commercial or financial relationships that could be construed as a potential conflict of interest.

## Footnotes

^1^ClinicalTrials.gov (accessed January 1, 2020).
