# Supplementary material for: Cross-Sectional Survey of Clinical Trials of Stem Cell Therapy for Heart Disease Registered at ClinicalTrials.gov
Source: Front Cardiovasc Med. 2021 Jul 8;8:630231. doi: 10.3389/fcvm.2021.630231 (PMC8295466; doi:10.3389/fcvm.2021.630231)
Supplement: Supplementary file 1 [file Data_Sheet_1.ZIP › 630231/stem cell and heart disease assignment.docx]

# assignment：

Status：

| Active, not recruiting 1 | Recruiting or rolling by invitation 2 | Suspended 3 |
| --- | --- | --- |
| Terminated 4 | Completed 5 | Unknown status 6 |
| Withdrawn 7 |  |  |

Withdrawn

| Contract issues 1 | no participants enrolled 2 |
| --- | --- |
| Study never started 3 | Funding not obtained and no participants enrolled 4 |
| inability to recruit due to administrative difficulties at the site 5 | Corporate business decision, may consider different subject population 6 |
| Unknown 7 | company dissolved 8 |
|  |  |
|  |  |

Results:

| has results 1 | No Results Available 2 |
| --- | --- |

Conditions:

| Heart Failure 1 | Coronary Artery Disease 2 | Cardiomyopathy 3 |
| --- | --- | --- |
| CHD 4 | Rhythmic disease 5 | Valvular disease 6 |
| others 7 | Hypertension or Arterial Hypertension 8 |  |
|  |  |  |
|  |  |  |

Gender:

| all 1 | male 2 | female 3 |
| --- | --- | --- |
| unknown 4 |  |  |

Age group:

| child | Y 1 | 2 |
| --- | --- | --- |
| adult | 1 | 2 |
| Old adult | 1 | 2 |
| Min-age max-age |  |  |
| Child 1 | Adult 2 | Old adult 3 |
| Child, adult 4 | Adult, old adult 5 | All 6 |

Phase

| Phase1 1 | Phase 2 2 |
| --- | --- |
| Phase3 3 | Phase4 4 |
| Phase1 and 2 5 | Phase 2\|Phase 3 6 |
| Not Applicable or unknown 7 |  |

Fund by

| US fed 1 | Us fed and others 2 | NIH 3 |
| --- | --- | --- |
| NIH and other 4 | NIH and Industry 5 | Industry 6 |
| Industry\|Other 7 | Industry\|NIH\|Other 8 | Other 9 |

Study type

| Interventional 1 | Observational 2 |  |
| --- | --- | --- |

United StatesStudy design/allocation

| Allocation: Randomized 1 | Other 2 | Not Applicable 3 |
| --- | --- | --- |

Study design/ interventional model/ Observational Model

| Parallel Assignment 1 | Single Group Assignment 2 | Crossover Assignment 3 |
| --- | --- | --- |
| Factorial Assignment 4 | Sequential Assignment 5 | Case-Control 6 |
| Cohort 7 | Other 8 | Not Applicable or unknown 9 |

Study design/Mask

| Single 1 | Double 2 | Triple 3 |
| --- | --- | --- |
| Quadruple 4 | None 5 | Not Applicable or unknown 6 |

Study Designs/Primary Purpose

| Treatment 1 | Diagnostic 2 | Basic Science 3 |
| --- | --- | --- |
| Prevention 4 | Health Services Research 5 | Not Applicable or unknown 6 |

Start year

|  |  |  |
| --- | --- | --- |
|  |  |  |
|  |  |  |
|  |  |  |

Location

| United States 1 | Denmark 2 | Japan 3 |
| --- | --- | --- |
| Italy 4 | China 5 | Greece 6 |
| Mexico 7 | Germany 8 | India 9 |
| France 10 | Slovenia 11 | Brazil 12 |
| United Kingdom 13 | Australia 14 | Singapore 15 |
| Iran 16 | Belgium 17 | Canada 18 |
| Spain 19 | Korea 20 | Russian 21 |
| Poland 22 | Israel 23 | Indonesia 24 |
| Ukraine 25 | Austria 26 | Finland 27 |
| Colombia 28 | Kazakhstan 29 | Malaysia 30 |
| Chile 31 | Norway 32 | Switzerland 33 |
| Turkey 34 | Unknown 35 |  |

Publications

| YES1 | NO 2 |
| --- | --- |
|  |  |
|  |  |

Study type

| Basic research 1 | Stem cell 2 | Non-stem cell 3 |
| --- | --- | --- |
| Abnormal 4 | Not Applicable |  |
|  |  |  |

Efficacy

| Yes 1 | No 2 | Not assessment 3 |
| --- | --- | --- |

Safety

| Yes 1 | No 2 | Not assessment 3 |
| --- | --- | --- |
